# Construct validity and test–retest reliability of the International Fitness Scale (IFIS) in Colombian children and adolescents aged 9–17.9 years: the FUPRECOL study

**DOI:** 10.7717/peerj.3351

**Published:** 2017-05-23

**Authors:** Robinson Ramírez-Vélez, Sandra Milena Cruz-Salazar, Myriam Martínez, Eduardo L. Cadore, Alicia M. Alonso-Martinez, Jorge E. Correa-Bautista, Mikel Izquierdo, Francisco B. Ortega, Antonio García-Hermoso

**Affiliations:** 1 Centro de Estudios en Medición de la Actividad Física (CEMA), Universidad del Rosario, Bogota, Cundinamarca, Colombia; 2 Exercise Research Laboratory, Physical Education School, Federal University of Rio Grande do Sul, Porto Alegre, Recife, Brazil; 3 Department of Health Sciences, Public University of Navarra, Pamplona, Navarra, Spain; 4 PROFITH “PROmoting FITness and Health Through Physical Activity” Research Group, Department of Physical Education and Sports, Faculty of Sport Sciences, University of Granada, Granada, Andalucia, Spain; 5 Department of Biosciences and Nutrition at NOVUM, Karolinska Institute, Huddinge, Sweden; 6 Laboratorio de Ciencias de la Actividad Física, el Deporte y la Salud, Facultad de Ciencias Médicas, Universidad de Santiago de Chile, Santiago, Chile

**Keywords:** Child, Physical fitness, Surveys and questionnaires, Risk factors, Self-report

## Abstract

**Background:**

There is a lack of instruments and studies written in Spanish evaluating physical fitness, impeding the determination of the current status of this important health indicator in the Latin population, especially in Colombia. The aim of the study was two-fold: to examine the validity of the International Fitness Scale (IFIS) with a population-based sample of schoolchildren from Bogota, Colombia and to examine the reliability of the IFIS with children and adolescents from Engativa, Colombia.

**Methods:**

The sample comprised 1,873 Colombian youths (54.5% girls) aged 9–17.9 years. We measured their adiposity markers (waist-to-height ratio, skinfold thickness, percentage of body fat and body mass index), blood pressure, lipids profile, fasting glucose, and physical fitness level (self-reported and measured). A validated cardiometabolic risk index score was also used. An age- and sex-matched subsample of 229 schoolchildren who were not originally included in the sample completed the IFIS twice for reliability purposes.

**Results:**

Our data suggest that both measured and self-reported overall physical fitness levels were inversely associated with percentage of body fat indicators and the cardiometabolic risk index score. Overall, schoolchildren who self-reported “good” or “very good” fitness had better measured fitness levels than those who reported “very poor/poor” fitness (all *p* < 0.001). The test-retest reliability of the IFIS items was also good, with an average weighted kappa of 0.811.

**Discussion:**

Our findings suggest that self-reported fitness, as assessed by the IFIS, is a valid, reliable, and health-related measure. Furthermore, it can be a good alternative for future use in large studies with Latin schoolchildren from Colombia.

## Introduction

Physical fitness is a state of being that includes skill- and health-related to present and future health outcomes. In particular, cardiorespiratory fitness (CRF), muscular fitness (MF) (e.g., muscle endurance, muscle strength, muscle power), and motor fitness (e.g., balance, coordination) can be immensely influenced by lifestyle factors (Ortega et al., 2008). Previous large cohort studies have shown that a lack of physical fitness (i.e., CRF) is an independent risk factor of cardiovascular disease (CVD) (LaMonte et al., 2005), even exceeding the influence of other classic factors of CVD such as dyslipidemia, hypertension, smoking and obesity (Lee et al., 2012; Swift et al., 2013). Similarly, MF in both men and women represents a different and independent predictor of cardiometabolic disease (Vaara et al., 2014) in young people and adolescents (Artero et al., 2011; Magnussen et al., 2012). Ruiz et al. (2009) reported a relationship between neuromotor fitness (i.e., muscle endurance, muscle strength, muscle power, speed, flexibility, agility, balance, coordination and reaction time) and health-related outcomes. In their study, physical fitness outcomes were positively related with blood pressure, and inversely related with adiposity markers, such as fat mass and abdominal obesity in children and adolescents (Ruiz et al., 2009).

Nevertheless, due either to the level of complexity involved in estimating physical fitness or the expense and difficulty associated with recruiting a qualified testing team and providing accessories, several authors have described easily-administered instruments that do not require sophisticated technology and are/have been validated by self-report scales or questionnaires, such as the five-level activity index (PAI) (Jackson et al., 2012). Researchers working on the Healthy Lifestyle in Europe by Nutrition in Adolescence (HELENA) study developed a self-report questionnaire regarding physical fitness questionnaire called the International Fitness Scale (IFIS) (Ortega et al., 2011). This scale has been validated in adolescents from nine European countries (Ortega et al., 2011), in young adults (18–30 years of age) (Ortega et al., 2013) and children (9–12 years of age) in Spain (Sánchez-López et al., 2015). Moreover, the IFIS has been shown to be strongly associated with CVD risk factors such as body fat or metabolic syndrome (Ortega et al., 2011; Ortega et al., 2013), even in Spanish women with fibromyalgia (Álvarez-Gallardo et al., 2016). Therefore, it appears to be a valid and reliable instrument to measure skill- and health-related physical fitness levels in several populations.

However, there is a lack of instruments and studies written in Spanish evaluating physical fitness, which hampers the determination of the current status of this important health indicator in the Latin population, especially in Colombia. Although the studies noted above have confirmed the validity of the IFIS with European populations, the authors claimed that there is a need to investigate the validity and reliability of this instrument when measuring self-reported physical fitness in other countries and populations (Español-Moya & Ramírez-Vélez, 2014). To the best of our knowledge, no study has evaluated the validity and reliability of the IFIS outside Europe. Therefore, the present study expands upon the knowledge regarding the application of the IFIS in Latin populations (Ramírez-Vélez et al., 2015). The aim of the study was two-fold: to examine the validity of the IFIS with a population-based sample of schoolchildren from Bogota, Colombia and to examine the reliability of the IFIS with children and adolescents from Engativa, Colombia.

## Methods

### Study design

Schoolchildren included in this secondary analysis are part of The Fuprecol Study (*Asociación de la Fuerza Prensil con Manifestaciones Tempranas de Riesgo Cardiovascular en Niños y Adolescentes Colombianos* in Spanish) carried out in Bogotá, Colombia. We selected 27 schools, which had already collaboration agreements established with our research center and therefore were selected primarily for pragmatic, budgetary and logistical reasons. The Fuprecol Study methodology has been published elsewhere (Ramírez-Vélez et al., 2015; Prieto-Benavides, Correa-Bautista & R., 2014; Rodríguez-Bautista et al., 2014). Data were collected from 2013 to 2016 and the analysis was done in 2016.

### Study population

The subsample consisted of 2,144 schoolchildren, a subsample of the FUPRECOL study. In this sample, 1,873 schoolchildren (54.5% girls) had valid data from the IFIS and anthropometric and blood parameter assessments; consequently, they were included in this study. The exclusion criteria included having a clinical diagnosis of cardiovascular disease, having Type 1 or Type 2 diabetes mellitus, being pregnant, using alcohol or drugs, and not having lived in Bogota for at least one school year. Exclusion from the study was made effective a posteriori, without the students being aware of their exclusion, to avoid any undesired situations.

### Sample of the reliability study (Engativa, Bogota)

The test-retest study was conducted with a separate age-matched sample of Colombian children and adolescents from the Engativa district, which is located north of Bogota. Based on the minimum size required for validity studies and assuming an attrition rate of 30%, a sample of at least 100 boys and 100 girls was randomly selected from the FUPRECOL study. The sample size was also satisfactory, according to the following formula: *n* = [(*Z*_α∕2_ + *Z*_β_)∕[*F*(*Z*_0_) + *F*(*Z*_1_)]]^2^ + 3. *α* = 0.05 (2-side), *β* = 0.1, *Z*_0.1_ = 1.282, *F*(*Z*_0_) = 0 and *F*(*Z*_1_) = 0.40, (Lemeshow et al., 1990).

A total of 229 participants (124 boys and 105 girls) aged 9–17.9 years successfully completed the IFIS on two occasions (one week apart) and were included in the reliability study. In addition, after the retest of the IFIS, physical fitness was measured in this sample using a battery of tests from the FUPRECOL study. The sample size was adequate for both validation and reliability, according to the estimations presented in several studies (Ortega et al., 2013; Sánchez-López et al., 2015; Álvarez-Gallardo et al., 2016). The characteristics of this sample did not differ from those of the FUPRECOL study cohort regarding SES or the inclusion and exclusion criteria. Finally, the same methods were used and investigators employed the IFIS in both studies.

### Measurements

#### Self-reported fitness

Self-reported fitness was assessed using the IFIS, which was originally validated in nine European countries and languages (HELENA study) (Ortega et al., 2011) (http://www.helenastudy.com/IFIS). The IFIS is comprised of 5 Likert-scale questions about self-reported fitness (very poor, poor, average, good, and very good) relating to perceived overall fitness and its main components: CRF, MF, speed and agility, and flexibility. In Colombia, the IFIS had high validity and moderate-to-good reliability in a study with collegiate students (Español-Moya & Ramírez-Vélez, 2014; Sánchez-López et al., 2015) and schoolchildren (Ortega et al., 2011) in European adolescents. All the information about the IFIS can be found at no cost at the website of the PROFITH research group, the original developer of this tool: http://profith.ugr.es/IFIS.

#### Anthropometric and adiposity variables

Data on the variables were collected at the same time in the morning (between 7:00 a.m. and 10:00 a.m.) following an overnight fast in accordance with the ISAK (International Society for the Advancement of Kinanthropometry) guidelines (Marfell-Jones, Stewart & De Ridder, 2006). Body weight was measured to the nearest 0.10 kg with the participant lightly dressed using a portable electronic weight scale (Tanita^®^ BC544; Tanita, Tokyo, Japan) with a low technical error of measurement (TEM = 0.510). Body height was measured to the nearest 0.1 cm in bare or stocking feet with the adolescent standing upright against a portable stadiometer (Seca^®^ 274; Seca, Hamburg, Germany; TEM = 0.019). Their BMI was calculated as their body weight in kilograms divided by the square of their height in meters (Ruiz et al., 2009). Body fat percentage was measured by bioelectrical impedance with a frequency current of 50 kHz using a BIA-TANITA_^®^_ Model BF689 (Tanita, Tokyo, Japan; TEM = 0.639) according to the manufacturer’s instructions. The mean of the two readings taken in the morning under controlled temperature and humidity conditions and after urination and a 15-minute rest when the participants was shoeless and fasting, was used.

#### Physical fitness

The physical fitness parameters were measured as described previously, and specific aspects relating to the validity and reliability have been reported elsewhere (Ramírez-Vélez et al., 2015; Español-Moya & Ramírez-Vélez, 2014). CRF was assessed using the 20-minute shuttle run test (Leger et al., 1988), and the time that it took to complete the last one-half stage (in minutes) was recorded. Results were recorded to the nearest stage completed. The Léger equation was used to determine VO_2_max (ml/kg/min) in each participant (Leger et al., 1988).

Musculoskeletal fitness (MF) was assessed using two tests. The standing broad jump (lower limb explosive strength assessment) was performed twice, and the best score was recorded (in kg). Additionally, the handgrip strength test (upper-body musculoskeletal strength) was performed using the T-18 TKK SMEDLY III^®^ standard adjustable handle analogue handgrip dynamometer (Takei Scientific Instruments Co., Ltd, Niigata, Japan). Two trials were allowed in each hand, and the average score was recorded (in kg). Thus, the values of handgrip strength that are presented here combined the results of left- and right-handed subjects, without any consideration of hand dominance (España-Romero et al., 2010).

Speed and agility (speed of movement, coordination and agility assessment) were measured using the 4 × 10 shuttle run test. The time that it took to complete the test was recorded to the nearest tenth of a second.

Flexibility was measured according to the standard sit-and-reach test for range of movement (in cm).

#### Biochemical assessments

Blood samples were obtained from each subject early in the morning, following a 10-hour overnight fast. Before the samples were taken, the fasting condition was confirmed by the child and the parents. Blood samples were obtained from an antecubital vein, and analyses were subsequently completed within one day of collection. The cholesterol linked to high-density lipoproteins (HDL-c), glucose, triglycerides (TG) and total cholesterol were measured using colorimetric enzymatic methods with a Cardiocheck analyzer. The fraction of cholesterol linked to low-density lipoproteins (LDL-c) was calculated using the Friedewald formula (Friedewald, Levy & Fredrickson, 1972). The precision performance of these assays was within the manufacturer’s specifications.

#### Cardiometabolic risk assessment

We calculated a cardiometabolic risk index (CMRI) as that reflects a continuous score of the five metabolic syndrome risk factors. An age-adjusted CMRI (composite *z*-score) was calculated for each participant using the following formula: CMRI = z-WC +z-triglycerides +z-HDL-C +z-glucose+z-SBP +zDBP. The HDL-c value was then multiplied by −1, as it is inversely related to cardiovascular risk.

The components of the score were selected based on the International Diabetes Federation’s (Zimmet et al., 2007) and the modified De Ferranti et al.’s (2004) definitions of metabolic syndrome (MetS). The higher the CMRI value, the higher the cardiovascular risk. All cut-off values were based on data that were obtained from schoolchildren internationally (Chan et al., 2015; Cruz et al., 2004; Suárez-Ortegón et al., 2012).

#### Sexual maturation

Participants self-assessed their sexual maturation of secondary sex characteristics (breast and pubic hair development for girls, genital and pubic hair development for boys; ranging from stage I to V), according to the criteria of Tanner & Whitehouse (1976) and Matsudo & Matsudo (1994). The data were recorded on paper by the FUPRECOL evaluators.

### Ethics statement

The Fuprecol Study was conducted in accordance with the Helsinki Declaration for Human Studies and approved by the Colombian Data Protection Authority (Resolution 008430/1993 Ministry of Health) and the Review Committee for Research on Human Subjects at the University of Rosario (Code No CEI-ABN026-000262). All participants were informed of the study’s goals, and written informed consent was obtained from participants and their parents or legal guardians.

### Statistical analysis

The test-retest reliability of the IFIS was indicated by the weighted *kappa* coefficient, which is more appropriate when dealing with ordered categorical data (Cohen, 1968). The internal consistency of the scales was assessed by calculating the Cronbach’s alpha. The ratings system developed by Landis & Koch (1977) was used to interpret the reliability and internal consistency results, where values ranging from 0.81 to 1.00 represent almost perfect agreement/consistency, 0.61 to 0.80 represent substantial agreement/consistency, 0.41 to 0.60 represent moderate agreement/consistency, 0.21 to 0.40 represent fair agreement/consistency, 0.00 to 0.20 represent slight agreement/consistency, and <0.00 represent poor agreement/consistency. The ability of the IFIS to rank Colombian youths into appropriate physical fitness levels correctly was determined using analysis of variance without any adjustment and then after adjusting (analysis of covariance (ANCOVA)) for sex, age and sexual maturation. Measured fitness variables were entered as dependent variables, whereas self-reported fitness variables were entered as fixed factors. We studied the association between self-reported fitness and both body fat and CMRI using ANCOVA after adjusting for sex, age and sexual maturation. Pairwise post hoc hypotheses were tested using the Bonferroni correction for multiple comparisons using four categories (very poor/poor, average, good and very good). The number of MetS criteria was calculated for each physical fitness component and its categories using the Jonckheere–Terpstra test for trends. All the analyses were conducted using IBM SPSS 21 (SPSS, Inc., Chicago, IL, USA). The level of statistical significance was set at  *p* < 0.05.

## Results

**Figure 1:**
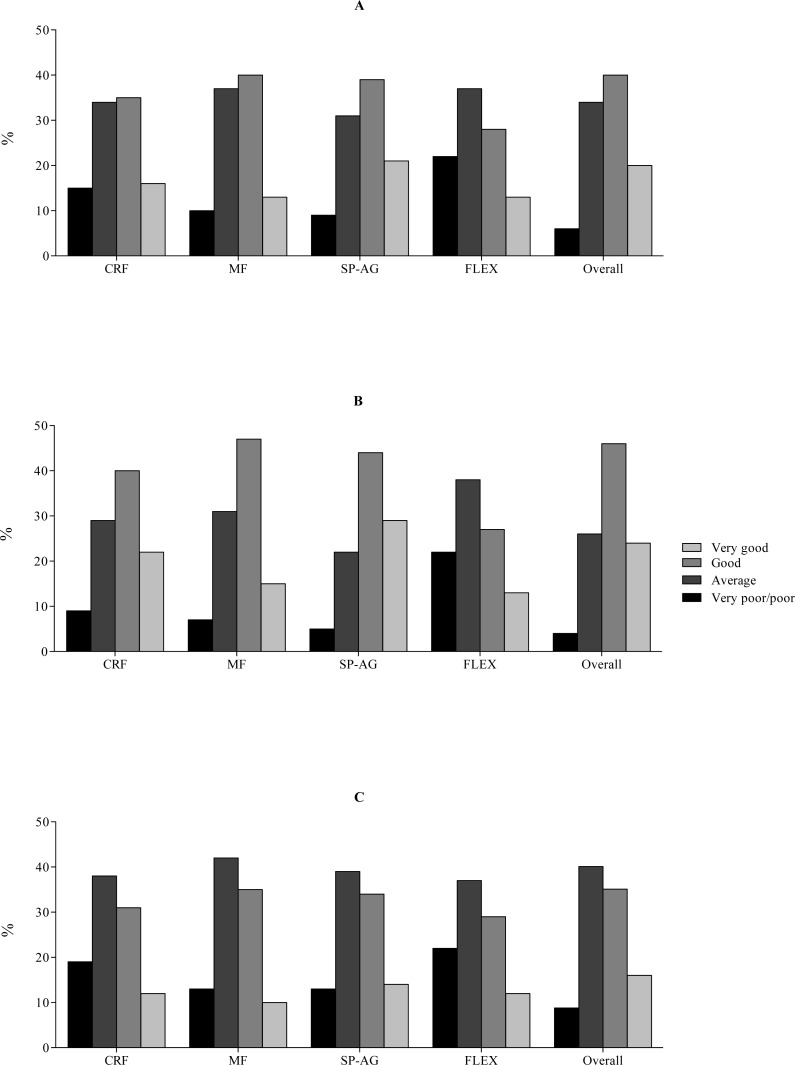
Distribution of the responses to the five questions of the International FItness Scale (IFIS) of schoolchildren in Bogota, Colombia. CRF, cardiorespiratory fitness; MF, muscular fitness; SP-AG, speed and agility; FLEX, flexibility; and Overall, overall physical fitness (A) Oveall; (B) Boys; (C) Girls.

The distribution of the self-reported responses on the IFIS using a 5-point Likert scale was shifted to the right in both genders, with a low percentage of participants reporting a very poor/poor fitness level (Fig. 1). Specifically, poor overall fitness was reported by 2% of the schoolchildren, whereas 4% reported poor, 34% reported average, 40% reported good, and 20% reported very good fitness. Participants who reported average, good, and very good CRF, musculoskeletal fitness, speed/agility, and flexibility had better measured CRF, MF, speed/agility, and flexibility compared to schoolchildren who reported a very poor/poor fitness level on the IFIS (Table 1).

**Table 1:** Unadjusted and adjusted means and standard error (SE) of measured physical fitness by self-reported physical fitness categories in Colombian children and adolescents, The FUPRECOL Study (*n* = 1, 873).

Components	Very poor/Poor (1) *n* = 119	Average (2) *n* = 618	Good (3) *n* = 756	Very good (4) *n* = 380	*P*-value	Pairwise comparisons^c^		
						1–2	2–3	3–4
Unadjusted								
20-m shuttle run (VO_2_max)	38.7 (0.3)	40.6 (0.2)	42.7 (0.1)	44.4 (0.2)	<0.001	<0.001	<0.001	<0.001
Handgrip (kg)	37.7 (0.5)	38.8 (0.2)	39.5 (0.2)	39.2 (0.4)	0.009	0.286	0.328	1.000
Standing-long jump (cm)	132.6 (2.1)	127.8 (1.1)	127.5 (0.9)	132.5 (1.8)	0.029	0.294	1.000	0.139
Shuttle run 4 ×10 m (s)^b^	15.3 (0.1)	14.9 (0.1)	14.3 (0.1)	14.1 (0.1)	<0.001	0.081	0.001	0.001
Sit and reach (cm)	20.1 (0.3)	20.6 (0.2)	21.3 (0.5)	22.5 (0.3)	<0.001	1.000	<0.001	0.355
Adjusted^a^								
20-m shuttle run (VO_2_max)	40.0 (0.4)	40.7 (0.1)	42.2 (0.1)	43.0 (0.2)	<0.001	0.790	<0.001	<0.001
Handgrip (kg)	37.8 (0.5)	38.9 (0.2)	39.4 (0.2)	39.3 (0.4)	0.050	0.042	1.000	1.000
Standing-long jump (cm)	136.6 (2.1)	127.9 (1.0)	127.3 (1.0)	132.0 (1.9)	0.036	0.318	1.000	0.183
Shuttle run 4 ×10 m (s)^b^	15.2 (0.1)	14.8 (0.1)	14.4 (0.1)	14.1 (0.1)	<0.001	0.068	<0.001	<0.001
Sit and reach (cm)	20.8 (0.3)	20.5 (0.2)	21.4 (0.5)	22.4 (0.3)	<0.001	1.000	<0.001	0.592

**Notes.**

a Analysis of covariance adjusted for sex, age and sexual maturation status.

b Lower scores indicating higher levels of speed-agility.

c Bonferroni-adjusted pairwise comparisons.

Table 2 shows the test-retest reliability statistics in children and adolescents from Engativa, Colombia for the five items that compose the IFIS, that is, overall fitness and four main fitness components: CRF, MF, speed and agility, and flexibility. The weighted kappa values ranged from 0.775 (handgrip) to 0.847 (standing long jump), and the average weighted *kappa* was 0.811.

**Table 2:** Test–retest (1 week apart) reliability of self-reported fitness in schoolchildren from Bogota, Colombia (*n* = 229).

IFIS components	Test mean (SD)	Re-test mean (SD)	Kappa	95% CI	α
Cardiorespiratory fitness	3.75 (0.81)	3.71 (0.85)	0.834	0.786–0.871	0.733
Muscular fitness	3.42 (0.97)	3.52 (0.97)	0.775	0.710–0.825	0.743
Speed and agility	3.54 (0.89)	3.55 (0.86)	0.847	0.803–0.881	0.763
Flexibility	3.78 (0.95)	3.7 (0.94)	0.797	0.739–0.842	0.726
Overall fitness	3.24 (1.00)	3.33 (1.01)	0.802	0.745–0.846	0.789

**Notes.**

IFIS International Fitness Scale SD standard deviation α Cronbach’s alpha

**Figure 2:**
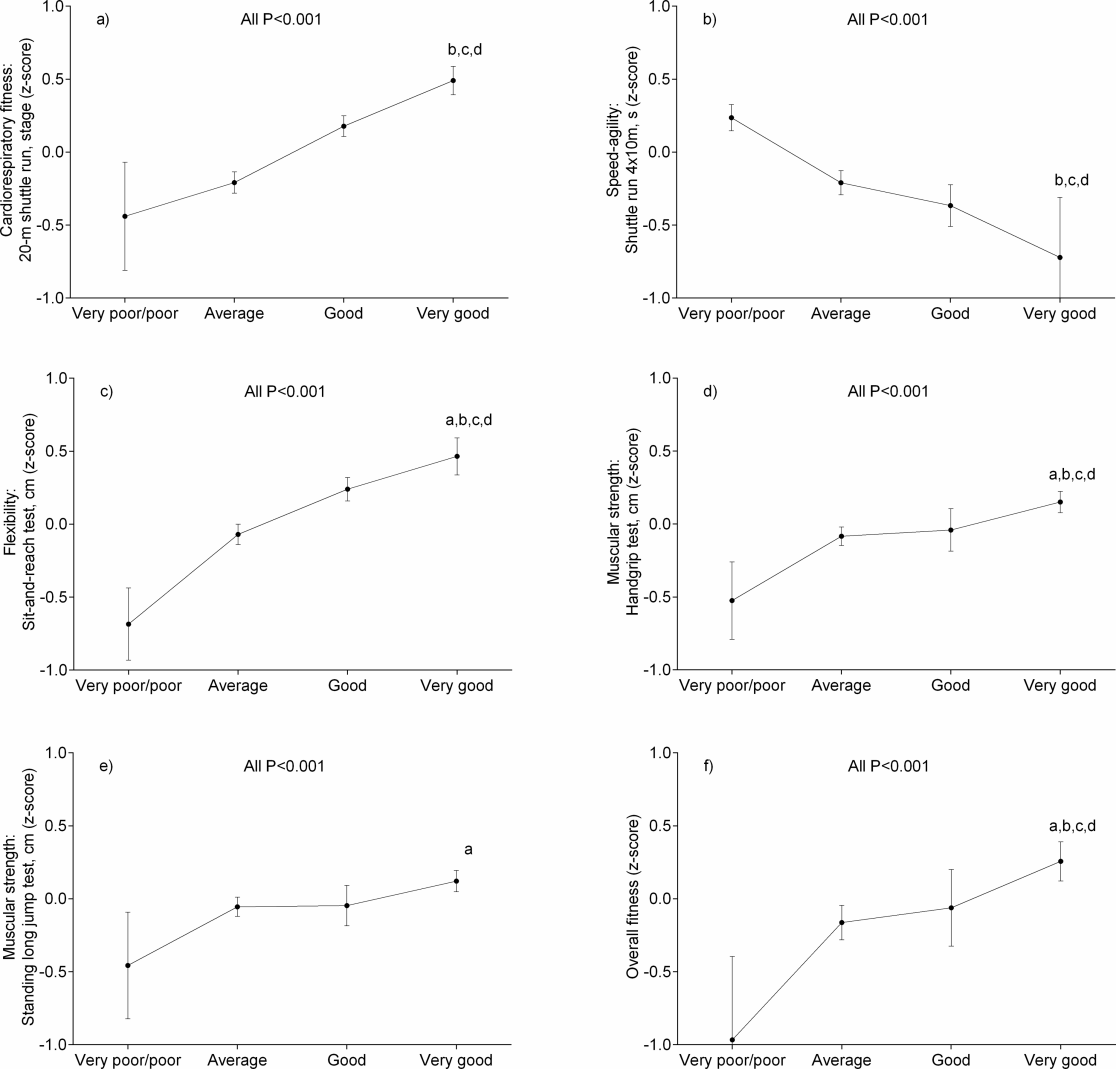
Associations between measured physical fitness and self-reported physical fitness categories in schoolchildren from Bogota, Colombia. Data represented means and 95% confidence intervals. Bonferroni-adjusted pairwise comparisons: (A) “good” vs “very good”; (B) “average” vs “very good”; (C) “very poor/poor” vs “very good”; (D) “very poor/poor” vs “very good”. Data represented means and 95% confidence intervals. All significance levels were *p* < 0.001.

The relationships between self-reported and measured variables are show in Fig. 2. Overall, participants with a high level of self-reported fitness (i.e., good and very good categories) had higher measured CRF, MF, speed and agility, and flexibility compared to schoolchildren reporting very poor/poor fitness level (all *p* < 0.001).

**Figure 3:**
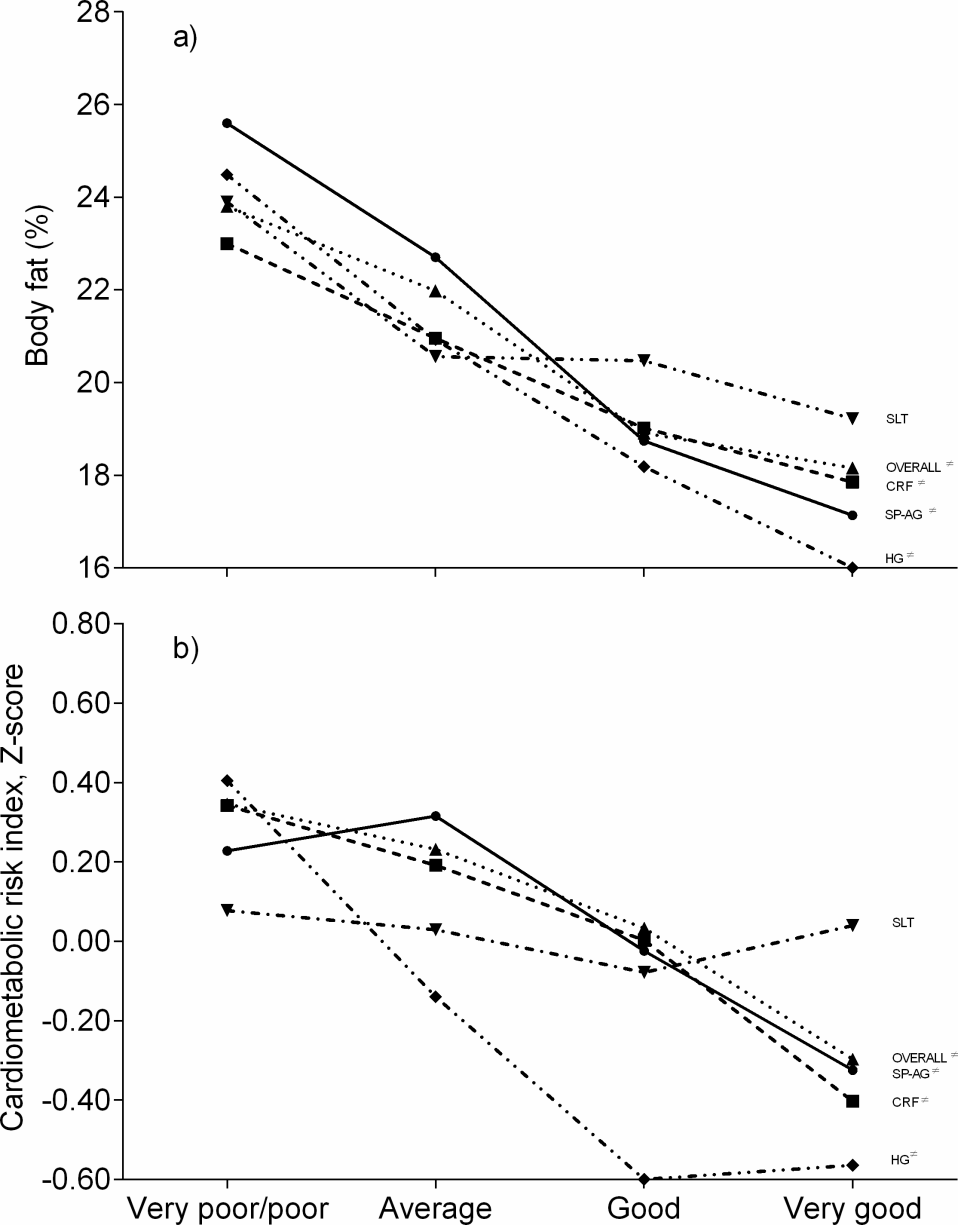
Associations between self-reported fitness, cardiometabolic risk index and body fat in schoolchildren from Bogota, Colombia. CRF, cardiorespiratory fitness; HG, handgrip strength; SLT, standing long jump test; SP-AG, speed and agility; Overall, overall physical fitness. ≠ Significance levels were *p* (trend) <0.001.

Figure 3 shows the association between self-reported fitness categories and CMRI and body fat. We observed an inverse association between body fat and CRF, MF, speed and agility, flexibility, and overall fitness (Fig. 3A). Finally, the CMRI score in Fig. 3B shows that a high level of self-reported CRF, MF, speed and agility, flexibility, and overall fitness is related to a lower CVD risk. For all the associations, the significance level was set at *p* < 0.05.

**Figure 4:**
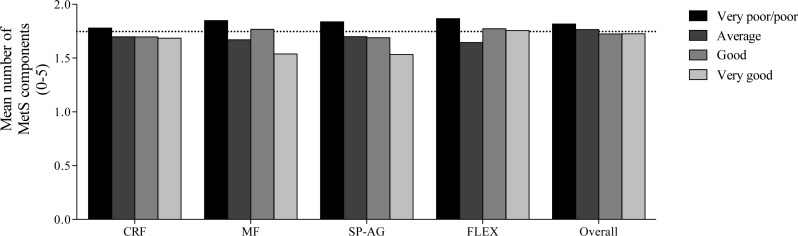
Trend distribution of number of metabolic syndrome components criteria (defined by De Ferranti et al., 2004) according to self-reported physical fitness components and its categories (the Jonckheere–Terpstra test). Horizontal line indicates the mean number of metabolic syndrome components in the overall population. CRF, cardiorespiratory fitness; MF, muscular fitness; SP-AG, speed and agility; and Overall, overall physical fitness.

Finally, Fig. 4 shows a dose–response relationship between self-reported physical fitness components, their categories and the number of MetS criteria (as defined by De Ferranti et al., 2004) in children and adolescents. As shown in the figure, a significant trend across categories was observed for all the components (*ps* < 0.001).

## Discussion

Our study shows that the IFIS had substantial validity and test-retest reliability for ranking schoolchildren according to their objectively measured health-related physical fitness. Additionally, self-reported CRF, MF, speed and agility, and flexibility as measured by the IFIS were negatively associated with lower CVD and adiposity risk in the schoolchildren who were examined.

Regarding the examination of the validity of the IFIS in Colombian schoolchildren, our results showed significant differences in the measured physical fitness components (CRF, MF, speed and agility, and flexibility) between schoolchildren reporting poor and very poor levels and those reporting average, good, and very good levels. These results are consistent with the studies of the HELENA project, which demonstrated that the IFIS has good validity in different populations (Ortega et al., 2011; Ortega et al., 2013; Sánchez-López et al., 2015). Results from the nine European countries showed that youths (3,528 adolescents aged 12.5–17.5 years) with good or very good physical fitness had better measured fitness compared to those self-reporting poor or very poor fitness on the IFIS; however, a linear dose–response relationship between self-reported and objectively measured physical fitness on the IFIS was observed (Ortega et al., 2011). In another study of 276 young adults (18–30 years of age), Ortega et al. (2013) observed that participants reporting good/very good CRF, musculoskeletal fitness and flexibility had better measured CRF, musculoskeletal fitness and flexibility compared to middle-aged adults reporting poor/very poor fitness.

Consistent with previous findings, there was a dose–response association between self-reported and measured CRF and flexibility, whereas the dose–response association between self-reported and measured MF was linear only when MF was expressed as an absolute value (Ortega et al., 2013). In addition, in Spanish children aged 9–12 years, Sánchez-López et al. (2015) observed that participants reporting average, good and very good CRF, MF, speed and agility, and flexibility had better performance cardiovascular endurance, musculoskeletal fitness, agility/speed and flexibility compared to those reporting very poor and poor physical fitness. Additionally, dose–response associations between self-reported and measured CRF, speed and agility, and flexibility as well as MF when expressed in absolute terms were observed (Sánchez-López et al., 2015). Moreover, even in specific populations such as women with fibromyalgia, the IFIS has shown good validity (Álvarez-Gallardo et al., 2016). The test-retest reliability of the IFIS observed in the present study revealed a weighted *kappa* ranging from 0.775 to 0.847 (average 0.811), which could be considered good agreement in our population. The reliability of the IFIS demonstrated in our study was higher than the test-retest coefficients observed in previous studies that analyzed Spanish children between nine and twelve years of age (average *kappa* = 0.70), adolescents from different European countries (*kappa* coefficients ranged from 0.54 to 0.65), Spanish young adults aged 18–30 years (average *kappa* = 0.70), and Spanish women with fibromyalgia (average *kappa* = 0.45). Differences across studies might be due to population characteristics; consequently, it would be interesting to replicate the study in other populations and perform future confirmatory studies to analyze the scale’s validity and reliability. Therefore, the present results suggest that the IFIS is a very reliable tool for use with Latin schoolchildren from Bogota, Colombia.

It has been shown that poor physical fitness (i.e., CRF) is an independent risk factor of CVD (LaMonte et al., 2005). In addition, it has been demonstrated that neuromotor fitness is associated with health outcomes such as systolic blood pressure and the sum of four skinfolds (Ruiz et al., 2009). The feasibility of conducting physical assessments to identify CVD risk factors is a crucial factor in environments in which time, equipment or qualified personnel might not be available (Ruiz et al., 2009). In this context, physical fitness questionnaires such as the IFIS might be useful, as self-reported fitness levels have been associated with CVD risk factors in different populations (Ortega et al., 2011; Ortega et al., 2013; Sánchez-López et al., 2015). Our study has shown that self-reported CRF, MF, speed and agility, flexibility, and overall fitness were inversely associated with CMRI and percentage of body fat, confirming previous studies of children (Ortega et al., 2011), young adults (Ortega et al., 2013), and adolescents (Sánchez-López et al., 2015) that also employed the IFIS. Sánchez-López et al. (2015) found that self-reported fitness levels were inversely associated with adiposity parameters (BMI, waist-to-height ratio and body fat-mass index). These authors also showed that CRF and speed and agility fitness levels were inversely associated with lower levels of total cholesterol, HDL cholesterol, triglycerides, HOMA, mean blood pressure and C-reactive protein. Taken together, these results suggest that the IFIS can be considered a useful instrument to investigate cardiometabolic risk using self-reported physical fitness indicators.

The strengths of the present study are: (i) this is one of the first studies on IFIS validity in a Latin population, explicitly describing the conceptual framework within which the IFIS was applied; and (ii) the present study has advanced the current knowledge regarding the validity and reliability of the IFIS, because we investigated these issues in a very large sample from a different country than those where the IFIS has been previously tested. However, the present study also has limitations. Although we investigated the validity and reliability of the IFIS in schoolchildren in a Latin country, more studies are needed for additional cross-validation testing in different ethnic groups of South America and other regions. In addition, the present data might have been affected by the average fitness level of the region, as all the schoolchildren assessed were recruited from the same region of Colombia. Although the instrument is not immune to the problems that are inherent in all self-report instruments such as their sensitivity to social prejudice, convenience and coherence, it has been shown that the IFIS is reliable in terms of estimating youth physical fitness.

## Conclusion

Our results have shown that the IFIS has validity when ranking Latin schoolchildren according to their directly measured health-related physical fitness. In addition, the present study has shown that the IFIS has good test-retest reliability in this population. Furthermore, a high level of self-reported fitness was associated with lower CVD and adiposity risk in children and adolescents from Bogota, Colombia.

## Additional Information and Declarations

### Data Availability

The following information was supplied regarding data availability: The raw data was made available for peer review but cannot be published due to legal restrictions and ethical imposed by the authors’ IRB (Uviversidad del Rosario (No CEI-ABN026-000262, Resolution 008430/1993 Ministry of Health). The subjects are children and adolescent students of Public Schools Colombia (Law No 1.581, October 2012 and National Decret No 1377 de 2013) and did not give consent to share their data. The authors are able to share a de-identified data set with other academic researchers who have obtained IRB approval from their institution to analyze these data. These researchers will be required to sign a data confidentiality and protection agreement.
